# Paracrine Kynurenic Pathway Activation in the Bone of Young Uremic Rats Can Antagonize Anabolic Effects of PTH on Bone Turnover and Strength through the Disruption of PTH-Dependent Molecular Signaling

**DOI:** 10.3390/ijms22126563

**Published:** 2021-06-18

**Authors:** Krystyna Pawlak, Beata Sieklucka, Dariusz Pawlak

**Affiliations:** 1 Department of Monitored Pharmacotherapy, Medical University of Bialystok, Mickiewicza 2C, 15-222 Bialystok, Poland; 2 Department of Pharmacodynamics, Medical University of Bialystok, Mickiewicza 2C, 15-222 Bialystok, Poland; beata.sieklucka@umb.edu.pl (B.S.); dariusz.pawlak@umb.edu.pl (D.P.)

**Keywords:** kynurenine pathway, parathyroid hormone (PTH), activating transcription factor 4/parathyroid hormone 1 receptor/cyclic adenosine monophosphate (PTH1R/ATF4/cAMP) axis, osteoblasts, osteoclasts, bone turnover, bone biomechanical properties

## Abstract

Secondary hyperparathyroidism and abnormalities in tryptophan (TRP) metabolism are commonly observed in chronic kidney disease (CKD). The present study aimed to establish potential interactions between endogenous parathyroid hormone (PTH) and activation of the bone kynurenine (KYN) pathway in relation to bone turnover and strength in young rats after one month (CKD-1) and three months (CKD-3) of experimental CKD. TRP, KYN, KYN/TRP ratio and bone turnover markers (BTMs) were measured in trabecular and cortical bone tissue. Expression of aryl hydrocarbon receptor (AhR) and the genes involved in osteogenesis was determined in femoral bone. Biomechanical testing of femoral diaphysis and femoral neck was also performed. Activation of the KYN pathway in trabecular bone during CKD development intensified the expression of genes related to osteogenesis, which led to a decrease in cyclic adenosine monophosphate (cAMP) and BTMs levels, resulting in a stiffer and mechanically weaker femoral neck. In contrast, reduction of the KYN pathway in cortical bone allowed to unblock the PTH-dependent anabolic activating transcription factor 4/parathyroid hormone 1 receptor (PTH1R/ATF4) axis, led to cAMP accumulation, better bone turnover and strength in the course of CKD development. In summary, the paracrine KYN pathway in bone can interfere with the anabolic effects of PTH on bone through disrupting PTH-dependent molecular signaling.

## 1. Introduction

Chronic kidney disease (CKD) is associated with the development of mineral bone disorder (MBD), leading to osteoporosis, bone fragility and increased risk of fracture [1,2]. Among CKD patients, low bone turnover is considered to be one of the most common types of renal osteodystrophy; it has been recognized both among CKD patients undergoing conservative treatment [3] and in patients on hemodialysis [4,5].

Bone turnover defines the rate of skeletal remodeling, reflected as the ratio between bone formation and bone resorption. Bone resorption is achieved by osteoclasts (OCs), after which the osteoblasts (OBs) form new bone matrix, leading to the restoration of the removed bone [6]. In physiological conditions, these two processes, which are referred to as coupling bone remodeling, are tightly balanced [7]. Since bone turnover in CKD is a function in large part of the degree of secondary hyperparathyroidism (SHPT), circulating parathyroid hormone (PTH) levels together with serum calcium, phosphorus and total alkaline phosphatase (ALP) activity are used to diagnose bone turnover and to guide the pharmacologic treatment of CKD-MBD [4]. However, SHPT is not always accompanied by high bone turnover [8]. During the early stages of the disease, patients with CKD often exhibit low bone turnover before transitioning to high bone turnover when SHPT is exacerbated. The variations in bone turnover are considered in part a function of abnormalities in PTH signaling [9], but the complex relationship between them during CKD is still poorly understood.

PTH affects both bone formation and resorption, and the activities of OCs and OBs are linked through the normal process of bone remodeling. Evidence from animal and clinical studies indicated that some aspects of the resorptive action of PTH are necessary for its anabolic effects in bone [10,11,12,13]. In addition to the direct effects of PTH on pre-osteoblasts, its transient effect has been observed rather on the promotion of OC activation than OC formation [14]. OC differentiation is supported by OBs expressing membrane-bound receptor activator of the NF-kB ligand (RANKL), and its secreted decoy receptor-osteoprotegerin (OPG) [15]. Previously, we found that serum PTH and the bone activating transcription factor 4/parathyroid hormone 1 receptor (PTH1R/ATF4) axis, the major pathway responsible for anabolic PTH effect in bone, can inversely affect the bone RANKL/OPG ratio and thus it may regulate bone accrual and strength in young rats with CKD [16].

Recently, tryptophan (TRP) metabolism through the kynurenine (KYN) pathway has been postulated as an important factor in promoting bone-aging phenotypes by interfering with stem cell function and the osteogenesis process [17]. El Refaey et al. [18] observed that feeding 12-month-old mice with kynurenine mimicked the bone aging process, resulting in bone parameters comparable to those of 24-month-old animals. However, knowledge on the impact of the KYN pathway activation on bone associated with CKD-MBD is very limited. Previously, in an experimental rat model, we showed that abnormalities in TRP metabolism have been implicated in disturbances of bone disease in CKD [19,20]. We also demonstrated that the endogenous KYN system in bone can regulate osteoblastogenesis and bone mineral status in an animal model of CKD treated with LP533401, an inhibitor of peripheral serotonin synthesis [21].

CKD-MBD in children causes long term consequences, such as growth failure, low peak bone mass and a tendency to fracture. However, the natural history of CKD—MBD in children is far from completely understood. The proper PTH level is needed for adequate bone remodeling, however the optimal PTH target range in children with CKD is not determined. Recent study of Soeiro et al. [22] demonstrated that in children with CKD low serum PTH levels (< 2 times of upper limit of normal) was independently associated with low bone turnover, loss of trabecular bone and abnormal mineralization. They also suggested that other variables might interfere in the relationship between PTH and bone parameters. Thus, the aim of the present study is to establish potential interactions between endogenous PTH and the KYN pathway activation in bone in relation to bone turnover and bone biomechanical properties in young, intensively growing rats with CKD.

The cross—sectional design of this study indicates associations that may exist and are therefore useful in generating hypotheses for future research. To prove causal relationships, the additional experimental investigations are needed under rationally controlled conditions.

## 2. Results

### 2.1. Paracrine Kynurenic System in Trabecular and Cortical Bone of Young Rats with CKD

**Figure 1:**
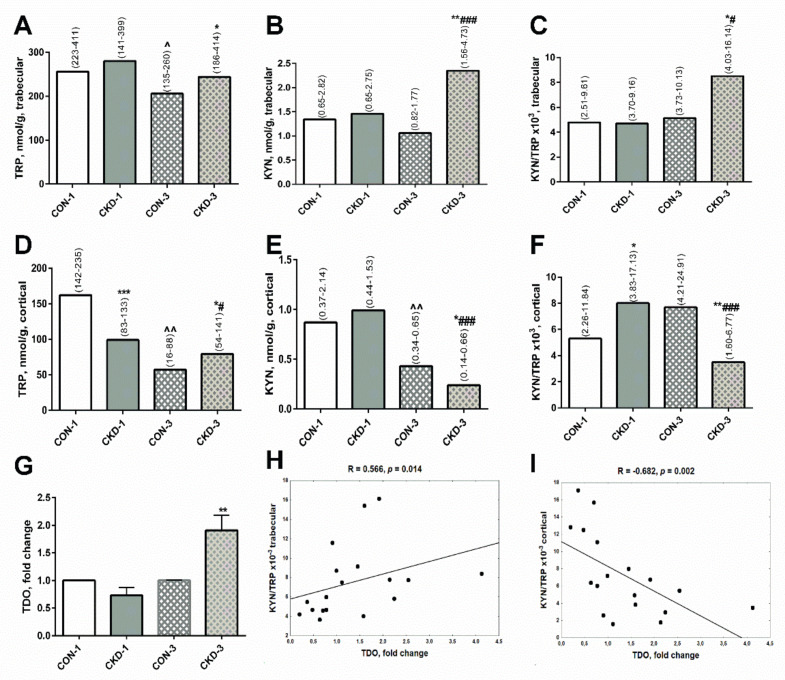
Kynurenine pathway in bone of young rats with chronic kidney disease (CKD). TRP (**A,D**), KYN (**B,E**) concentrations and KYN/TRP ratio (**C,F**) in bone homogenates, TDO gene expression in bone (**G**), and the association between TDO gene expression and KYN/TRP ratio in bone homogenates (**H,I**). Data are median (full range) or fold change ± s.e.m; * *p* < 0.05, ** *p* < 0.01, *** *p* < 0.001 controls versus appropriate CKD group; ^ *p* < 0.05, ^^ *p* < 0.01 CON-1 versus CON-3; # *p* < 0.05, ### *p* < 0.001 CKD-1 versus CKD-3; Abbreviations: CKD = chronic kidney disease; CON-1 = sham operated rats, 1 month after surgical intervention; CON–3 = sham operated rats, 3 month after surgical intervention; CKD-1 = subtotal nephrectomized rats, 1 month after surgical intervention; CKD—3 = subtotal nephrectomized rats, 3 month after surgical intervention; TRP = tryptophan; KYN = kynurenine; TDO = tryptophan 2,3—dioxygenase.

There was no difference in TRP, KYN levels and the KYN/TRP ratio in trabecular bone tissue the sham operated rats, 1 month after surgical intervention (CON-1) and subtotal nephrectomized rats, 1 month after surgical intervention (CKD-1) groups. During three months of CKD development (CKD-3), the concentrations of TRP, KYN as well as the KYN/TRP ratio were higher in uremic rats compared with appropriate controls (CON-3), and the activation of the KYN system was observed in these animals in comparison with the CKD-1 group (Figure 1A–C).

In cortical bone, TRP concentrations were significantly lower in CKD-1 rats compared with their healthy counterparts. Despite this, the KYN levels were similar between these groups, whereas KYN/TRP ratios, reflecting KYN system activation, were enhanced in the CKD—1 group compared with appropriate controls. Three months later, TRP and KYN concentrations gradually decreased both in the controls and the CKD animals. However, KYN levels and the KYN/TRP ratios were significantly lower in CKD-3 than in CON—3, reflecting a reduction of KYN system activation at this bone site (Figure 1D–F). 

Recently, we found that the activation of the paracrine KYN system in the bone of rats with CKD is tryptophan 2,3—dioxygenase (TDO)-dependent [21]. Herein, we measured TDO gene expression in femoral bone. As shown on Figure 1G, the expression of the TDO gene grew in rats with CKD-3 compared with CON-3, and it was associated with the KYN/TRP ratio (Figure 1H) and KYN levels (R = 0.370, NS) in trabecular bone. In contrast, strong inverse correlations were observed between TDO expression and the KYN/TRP ratio (Figure 1I) and KYN levels (R = −0.723, *p* = 0.0007) in cortical bone tissue.

### 2.2. Bone Turnover in Trabecular and Cortical Bone of Young Rats with CKD

**Figure 2:**
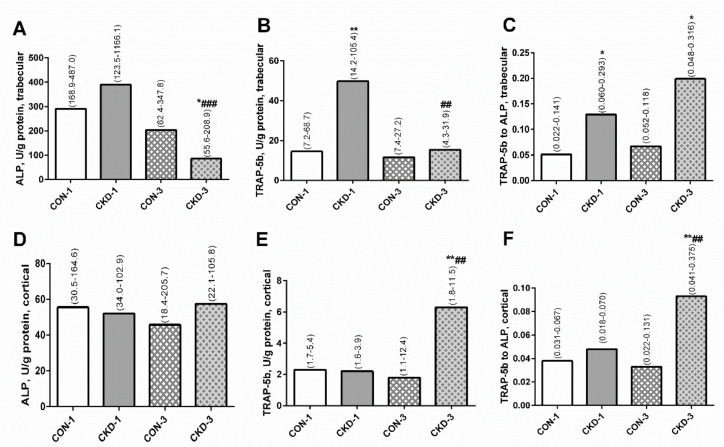
The bone turnover markers: ALP activity (**A,D**), TRAP-5b activity (**B,E**), and TRAP-5b to ALP ratio (**C,F**) in bone homogenates of young rats with chronic kidney disease (CKD). Data are median (full range). * *p* < 0.05, ** *p* < 0.01, controls versus appropriate CKD group; ## *p* < 0.01, ### *p* < 0.001 CKD-1 versus CKD-3; Abbreviations: ALP = alkaline phosphatase; CKD = chronic kidney disease; CON-1 = sham operated rats, 1 month after surgical intervention; CON-3 = sham operated rats, 3 month after surgical intervention; CKD-1 = subtotal nephrectomized rats, 1 month after surgical intervention; CKD-3 = subtotal nephrectomized rats, 3 month after surgical intervention; TRAP-5b = tartrate-resistant acid phosphatase 5b.

In homogenates of trabecular bone, ALP activity was similar in the CKD-1 group and in the corresponding controls (Figure 2A), whereas tartrate-resistant acid phosphatase 5b (TRAP-5b) activity and the TRAP-5b/ALP ratios were higher in the CKD-1 group compared with the CON-1 group (Figure 2B,C). During three months of CKD progression, there was a significant decrease in ALP activity in uremic animals compared with the CKD-1 group, and this enzyme activity was significantly lower in the CKD-3 group compared with the appropriate controls (Figure 2A). TRAP-5b activity was also significantly reduced in CKD-3 compared to CKD-1 animals, however it was similar between the CKD-3 and the CON-3 groups (Figure 2B). The TRAP—5b/ALP ratio remained higher in the CKD-3 group compared with the corresponding controls, which was caused by a significant decrease in ALP activity in the CKD-3 animals (Figure 2C). There was a strong, positive association between ALP and TRAP-5b activity (R = 0.742, *p* = 0.0001) in trabecular bone, indicating that bone formation was coupled with the resorption process at this bone level. In cortical bone tissue, the activity of ALP, TRAP—5b and the TRAP-5b/ALP ratios were similar between the CKD-1 and the CON-1 groups (Figure 2A–C). In the third month of CKD, TRAP—5b activity was significantly elevated compared with CKD-1 as well as CON-3 (Figure 2E), which resulted in an increased TRAP—5b/ALP ratio (Figure 2F). There was no relationship between ALP and TRAP—5b (R = 0.019, NS) in this bone region. Moreover, there was an inverse association between TRAP—5b activity in trabecular and cortical bone (R = −0.455, *p* = 0.038). This suggests that bone formation was not coupled with bone resorption in this bone region and that the resorption process in the trabecular bone ran independently of that in the cortical bone.

### 2.3. Association between Serum PTH, Bone KYN and Bone Turnover Markers (BTMs) in Young Rats with CKD

**Figure 3:**
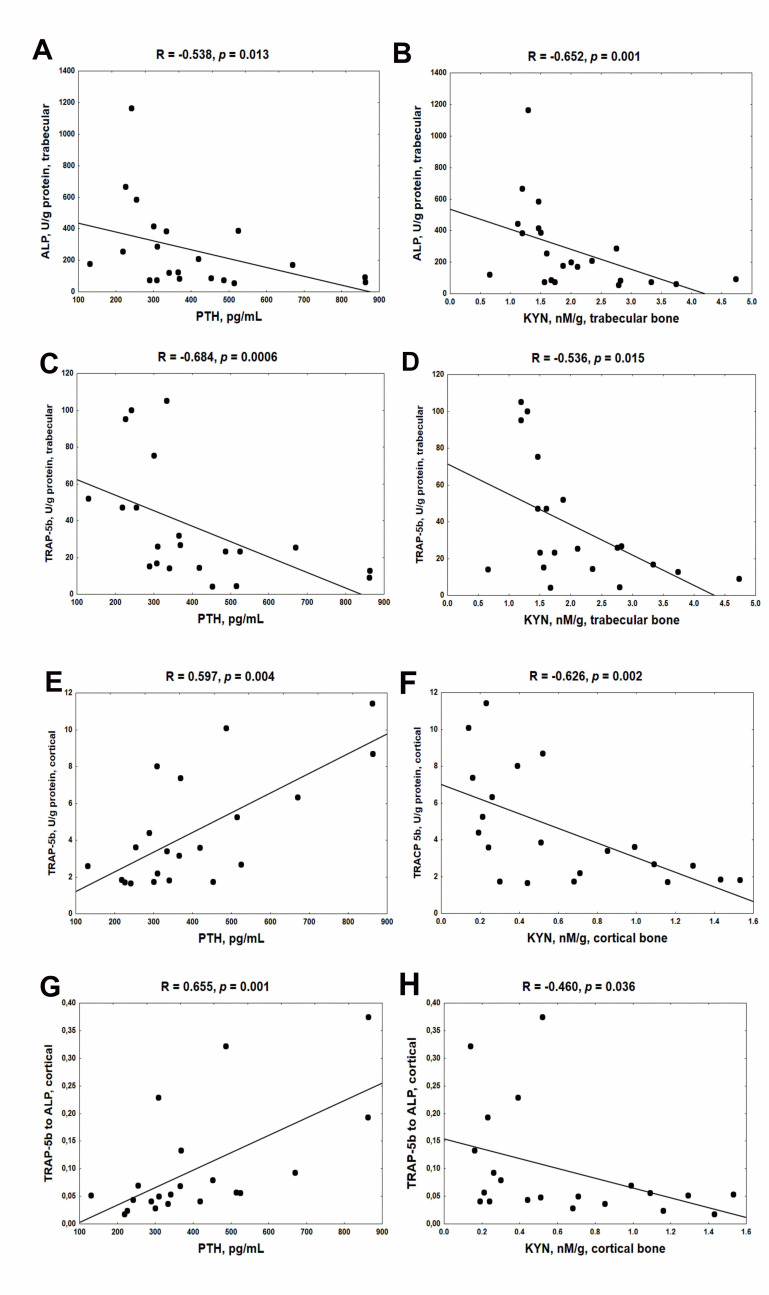
The association between bone turnover markers and serum PTH (**A,C,E,G**) and bone KYN (**B,D,F,H**) in young rats with chronic kidney disease (CKD). Abbreviations: ALP = alkaline phosphatase; CKD = chronic kidney disease; TRAP-5b = tartrate—resistant acid phosphatase 5b; PTH = parathyroid hormone; KYN = kynurenine.

Previously, we noticed that serum PTH levels were slightly but significantly increased in the third month of CKD compared with healthy animals (*p* < 0.05) [19]. As shown on the left side of Figure 3, endogenous PTH was inversely associated with ALP (Figure 3A), and particularly with TRAP-5b activity (Figure 3C) in trabecular bone. However, PTH strongly and positively correlated with both TRAP-5b (Figure 3E) and the TRAP-5b/ALP ratio (Figure 3G) in cortical bone tissue. On the right side of Figure 3, the associations between bone KYN and BTMs are shown. Independently from the analyzed bone region, KYN was inversely correlated with BTMs. These results indicate that endogenous PTH could attenuate bone turnover and especially bone resorption in trabecular bone, whereas it could amplify this process in cortical bone. Simultaneously, bone KYN seems to inhibit bone turnover irrespective of bone site.

### 2.4. Bone KYN Can Affect cyclic adenosine monophosphate (cAMP) Levels in Trabecular and Cortical Bone

**Figure 4:**
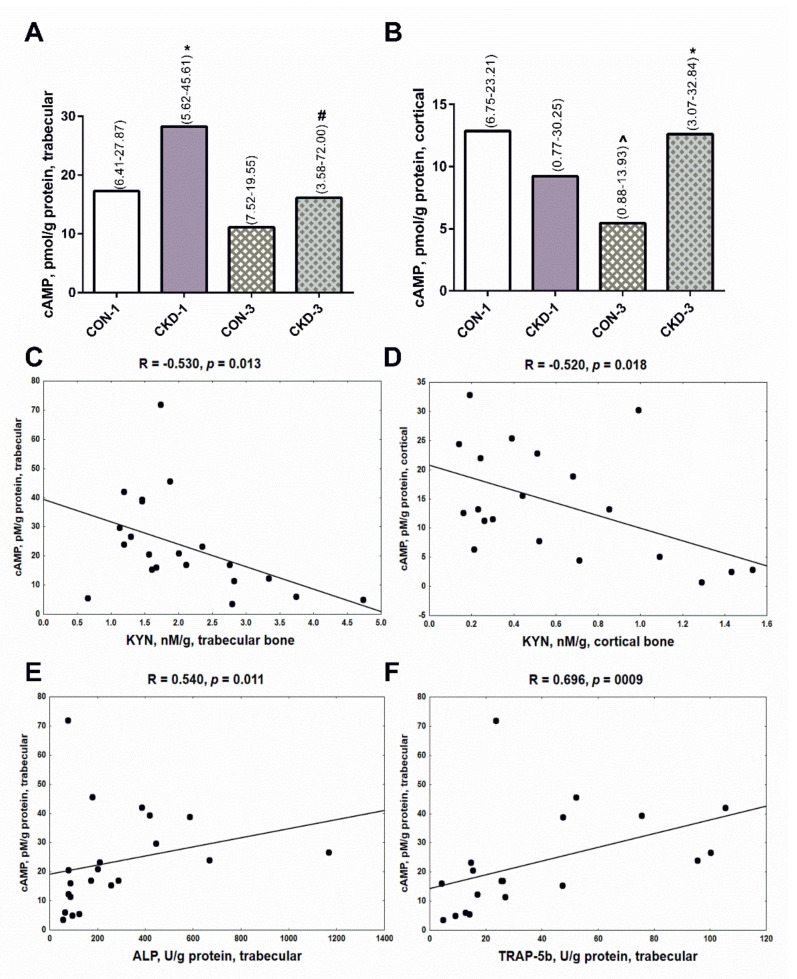
The concentrations of cAMP in bone of young rats with chronic kidney disease (CKD) (**A**,**B**), and its association with bone kynurenine (**C**,**D**) and bone turnover markers (**E**,**F**). Data are median (full range). * *p* < 0.05 controls versus appropriate CKD group; ^ *p* < 0.05 CON-1 versus CON-3; # *p* < 0.05 CKD-1 versus CKD-3; Abbreviations: cAMP = cyclic adenosine monophosphate; CON-1 = sham operated rats, 1 month after surgical intervention; CON-3 = sham operated rats, 3 month after surgical intervention; CKD-1 = subtotal nephrectomized rats, 1 month after surgical intervention; CKD-3 = subtotal nephrectomized rats, 3 month after surgical intervention; CKD = chronic kidney disease; KYN = kynurenine; ALP = alkaline phosphatase; TRAP-5b = tartrate-resistant acid phosphatase 5b.

The cAMP pathway is a major mediator of the anabolic action of PTH in bone [23,24], so we measured its concentrations in the analyzed bone regions. As shown in Figure 4A, cAMP levels in trabecular bone were higher in CKD-1 than in CON-1 animals, however they were significantly reduced in the CKD-3 group compared with the CKD-1 (Figure 4A). In cortical bone, cAMP levels were lower in CON-3 in comparison with CON-1, whereas this messenger was significantly elevated in rats with CKD-3 compared with appropriate controls (Figure 4B). Bone KYN levels were inversely related to cAMP concentrations both in trabecular (Figure 4C) and cortical (Figure 4D) tissue. We also noticed that cAMP was positively associated with the bone formation marker (Figure 4E), and especially with the bone resorption marker (Figure 4F) in trabecular bone. A tendency towards a positive association was also observed between cAMP and TRAP-5b (R = 0.377, *p* = 0.072) in cortical bone. These results suggest that bone KYN could modify cAMP production, and thus it could affect bone turnover, particularly in the trabecular bone region. 

### 2.5. Expression of Genes Involved in Osteoblasto- and Osteoclastogenesis Can Be Mediated by Both PTH1R and the aryl hydrocarbon receptor (AhR)-Dependent Pathway

**Figure 5:**
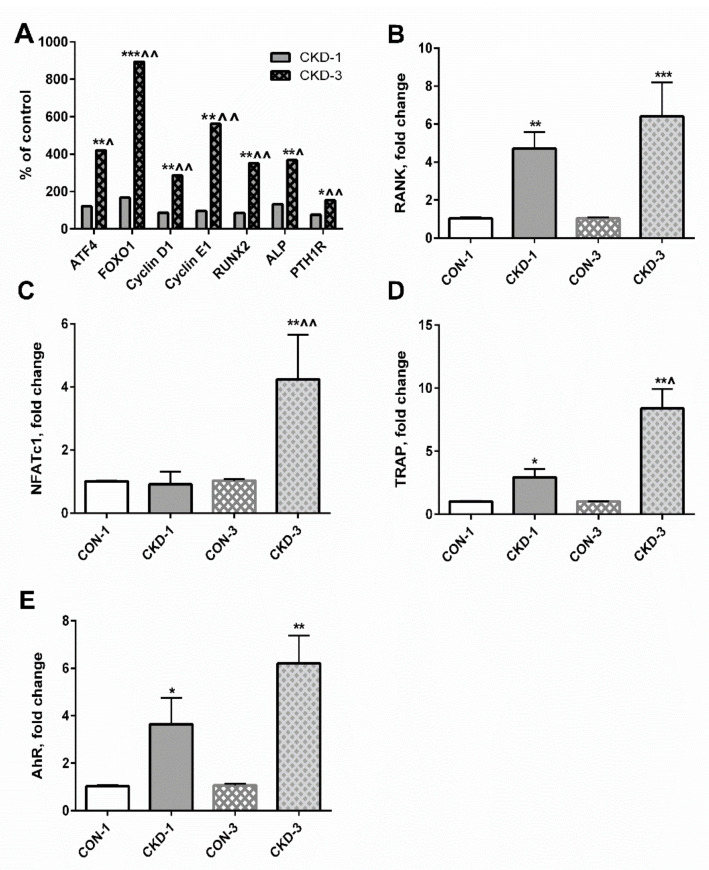
The expression of genes involved in osteogenesis (**A**–**D**), and the expression of aryl hydrocarbon receptor (AhR) gene (**E**) in bone of young rats with chronic kidney disease (CKD). * *p* < 0.05, ** *p* < 0.01, *** *p* < 0.001 CKD versus appropriate controls; ^ *p* < 0.05, ^^ *p* < 0.01 CKD-3 versus CKD-1. Abbreviations: ATF4 = activating transcription factor 4; CON-1 = sham operated rats, 1 month after surgical intervention; CON–3 = sham operated rats, 3 month after surgical intervention; CKD-1 = subtotal nephrectomized rats, 1 month after surgical intervention; CKD-3 = subtotal nephrectomized rats, 3 month after surgical intervention; CKD = chronic kidney disease; FOXO1 = Forkhead box transcription factor 1; RUNX2 = Runt-related transcription factor 2; ALP = alkaline phosphatase; PTH1R = parathyroid hormone 1 receptor; RANK = receptor activator of nuclear factor kappaB; NFATc = nuclear factor of activated T cell cytoplasmic 1; TRAP = tartrate—resistant acid phosphatase.

Previously, we determined the expression of numerous genes involved in osteoblastogenesis in young rats with CKD [25], the most important of which are schematically presented in Figure 5A. There were no differences in the expression of these genes between the CON-1 and the CKD-1 groups, however their expression was significantly elevated in CKD-3 compared with both CKD-1 as well as appropriate controls. In the present study, we measured the expression of key genes related to osteoclastogenesis in these groups. As presented in Figure 5B–D, the expression of the receptor activator of nuclear factor kappaB (RANK), the nuclear factor of activated T cell cytoplasmic 1 (NFATc1), and TRAP was significantly increased during CKD development. Similarly, the expression of AhR, which is now recognized as a major receptor for endogenous KYN signaling [26], gradually increased, starting from the first month of CKD compared with appropriate controls (Figure 5E). As shown in Table 1, the expression of PTH1R and AhR was strongly and positively associated with the expression of early genes of both osteoblasto- as well as osteoclastogenesis. However, PTH1R expression was particularly correlated with osteoblast differentiation markers, whereas AhR expression seems to be more involved in the osteoclastogenesis pathway. Moreover, there were close relationships between genes involved in osteoblastogenesis, as well as between RANK and genes involved in osteoblastogenesis (Table 2). Interestingly, RANK mRNA was not associated with the expression of the later markers of OCs differentiation—NFATc1 and TRAP—whereas a positive relationship existed between NFATc1 and TRAP gene expression.

### 2.6. Mechanistic Relationships between KYN Pathway Activation, Gene Expression, cAMP and BTMs in Bone Tissue of Young Rats with CKD

Both KYN levels as well as the KYN/TRP ratio in trabecular bone tissue were positively (or tended to be positively) associated with the expression of some analyzed genes, particularly with ATF4 and cyclin D1 (Table 3). However, the expression of these genes was inversely related to cAMP concentrations, ALP and TRAP-5b activity at this bone level.

**Figure 6:**
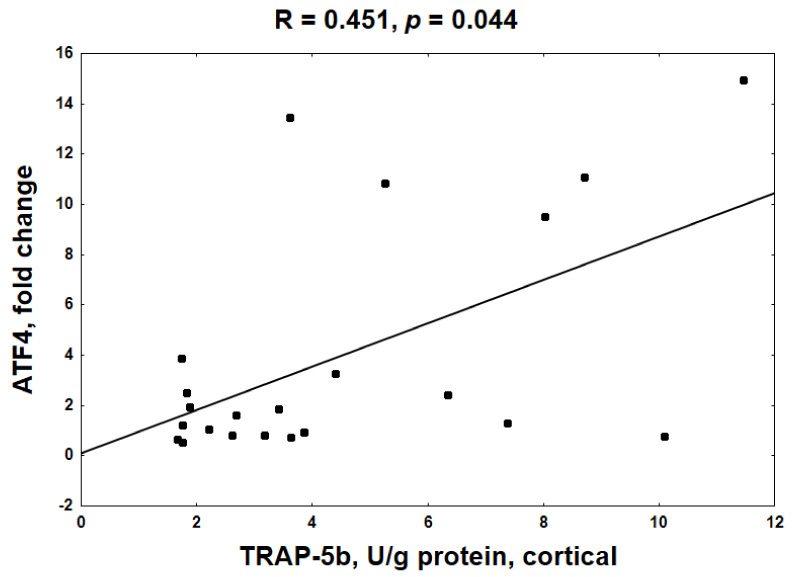
The relationship between the activating transcription factor 4 (ATF4) gene expression and tartrate-resistant acid phosphatase 5b (TRAP-5b) activity at cortical bone level.

In contrast to trabecular bone, there was no relationship between the decreased KYN system in cortical bone and the mRNA levels of the analyzed genes. However, a positive association between ATF4 gene expression and TRAP-5b activity (Figure 6) as well as ALP activity (R = 0.308, NS) was noted at this bone level. Moreover, positive, but not statistically significant, relationships also occurred between BTMs and the expression of the other analyzed genes (data not shown).

### 2.7. Endogenous Bone KYN and BTMs Can Affect the Biomechanical Properties of Trabecular and Cortical Bone of Young Rats with CKD

**Figure 7:**
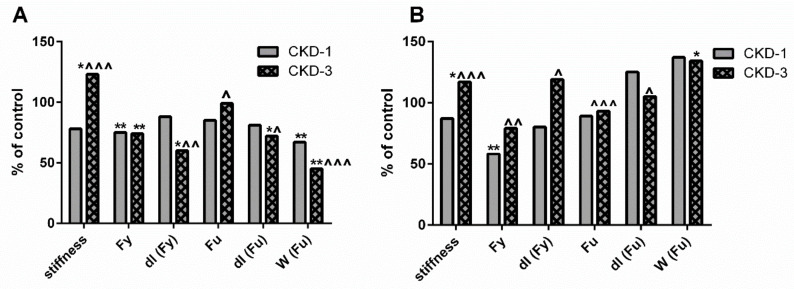
Schematic representation of the changes in biomechanical properties at trabecular (**A**) and cortical (**B**) level of femoral bone of young rats with chronic kidney disease (CKD). Data relate to control taken as 100%. * *p* < 0.05, ** *p* < 0.01 CKD versus appropriate controls; ^ *p* < 0.05, ^^ *p* < 0.01, ^^^ *p* < 0.001 CKD-3 versus CKD-1; Abbreviations: CKD = chronic kidney disease; CKD-1 = subtotal nephrectomized rats, 1 month after surgical intervention; CKD-3 = subtotal nephrectomized rats, 3 month after surgical intervention; Fy = yield load; dl (Fy) = displacement at yield load; Fu = ultimate load; dl (Fu) = displacement at ultimate load, W (Fu) = work to fracture.

Our previous results indicated that the biomechanical properties of cortical bone were preserved, whereas the biomechanical properties of trabecular bone were impaired compared with the healthy bone of young rats with a mild degree of CKD [19]. The biomechanical properties at the analyzed bone levels are presented schematically in Figure 7. In the present study, we studied the associations between the biomechanical parameters and bone KYN as well as bone turnover at appropriate bone regions. As presented in Table 4, a reduction in bone turnover was inversely associated with trabecular bone stiffness and ultimate load (Fu), while it was positively related to displacement at yield load [dl (Fy)]. Meanwhile, the opposite relations occurred between these parameters and trabecular KYN levels. 

At the cortical bone level, strong and inverse relationships were observed between decreased KYN values and the analyzed biomechanical bone properties, except for displacement at ultimate load [dl (Fu)], where a positive association with KYN was noticed. Interestingly, the majority of bone biomechanical properties were positively associated with TRAP-5b activity and the TRAP-5b/ALP ratio, but not with ALP activity at this bone level. Moreover, there was no connection between work to fracture [W (Fu)] and KYN levels, as well as between W (Fu) and BTMs in both analyzed bone regions (data not shown). 

## 3. Discussion

Bone, particularly in CKD progression, is considered one of the classical targets of PTH, as PTH1R expression is found in OBs, osteocytes as well as in OCs [27]. On the other hand, a detrimental effect of the KYN pathway on bone metabolism, especially in the elderly, has been recently suggested [28]. Since both SPHT and abnormalities in TRP metabolism are commonly observed in CKD, the potential interaction of these systems at the bone level and its significance for bone turnover and biomechanics were studied in the present work in young, growing rats with CKD.

Recently, we discovered the presence of a TDO-dependent paracrine kynurenic system in the bone of uremic 24-week-old rats with CKD [21]. The rats used in the present study were younger: 8-week-old (CKD-1) and 16-week-old (CKD-3) [19], but we also observed a TDO-dependent activation of the KYN pathway in their trabecular bone during three months of CKD development. In cortical bone, KYN system activation, reflected by an increased KYN/TRP ratio, was observed during the first month of CKD progression. However, a TRP deficiency at this bone level caused that KYN concentrations were similar between CKD-1 and appropriate controls (Figure 1E). During the three months of CKD development, there was a further reduction in TRP, and consequently in KYN levels, in both the uremic and the control groups. However, the lower KYN/TRP ratio in uremic animals indicated the inhibition of the KYN system in cortical bone, unlike the activation of this system observed in trabecular bone. These data demonstrated that TRP metabolism via the KYN pathway in juvenile CKD rats can be different depending on the analyzed bone level.

Bone formation and resorption are two continuous and interweaving processes that are part of bone remodeling. It is generally understood that the activities of OBs and OCs, through a dynamic equilibrium between them, are expected to ensure proper bone turnover for bone remodeling [7]. However, the underlying mechanism governing these cells’ behavior in CKD is still far from being fully understood. We observed that the resorption process dominated bone formation at the trabecular bone level, but despite this, these two processes were tightly coupled in rats with CKD. As shown on Figure 3, both trabecular KYN as well as serum PTH were inversely associated with BTMs, suggesting that KYN pathway activation and SPHT can inhibit bone turnover at this bone level. In cortical bone, strong stimulation of bone resorption, which was not coupled with bone formation, was seen in the CKD—3 group. Despite the fact that KYN remained in a reverse relationship with cortical resorption markers (Figure 3F,H), a positive association was seen between serum PTH and these markers (Figure 3E,G), indicating that cortical KYN and SPHT may have a different, opposite effect on the resorption process at this bone site.

The activation of PTH1R stimulates multiple downstream pathways, such as adenylate cyclase/cAMP/protein kinase A [29], which have been implicated as major mediators of the effects of PTH on bone remodeling. In this case, cAMP is a second messenger in the cellular action of PTH, and it is the major mediator of the anabolic action of this hormone in bone [23,24]. The results of our study showed the opposite effect of a 3-month CKD development on cAMP levels in the analyzed bone compartments (Figure 4A,B), which seems to be, at least partially, mediated by KYN levels in bone tissue (Figure 4C,D). So far, this is the only study showing that in vitro KYN can affect cAMP levels in human pulmonary arterial smooth muscle cells [30]. However, Kanatani et al. [31] reported that some compounds, such as estrogens via the estrogen receptor, can block cAMP-mediated OC formation stimulated by PTH in osteoclast precursors derived from mouse hemopoietic blast cells. These observations support our hypothesis that bone KYN could interfere with the cAMP—dependent action of PTH in bone. Importantly, cAMP levels were closely associated with BTMs, and especially with TRAP—5b, which reflects bone resorption. This finding indicates that the PTH/PTH1R/cAMP pathway is closely associated with bone turnover in young rats with CKD. A similar mechanism was previously reported by Sahbani et al. [32], who used PTH analogs, teriparatide and abaloparatide, to evaluate bone structure, microarchitecture and bone turnover in 16-week-old female mice. Taking the above together, it seems to be possible that KYN in trabecular bone, where its concentrations rose during CKD development, may antagonize the anabolic cAMP-dependent action of PTH, leading to reduced bone turnover.

In the next step of our study, we tried to identify the potential molecular mechanism by which KYN may affect osteogenesis in young animals with CKD. Previously [16,25], we showed that the expression of genes involved in osteoblastogenesis, as well as PTH1R mRNA levels, significantly increased during CKD progression, as schematically presented in Figure 5A. In the present study, we measured the expression of key genes associated with osteoclastogenesis—RANK, NFATc1 and TRAP. The activation of RANK on the surface of osteoclast progenitor cells leads to the induction of several downstream signaling molecules, including NFATc1, a master regulator of OCs differentiation, which regulates a number of osteoclast specific genes, among others TRAP [33]. The expression of these genes rose during CKD development, especially in the CKD-3 group (Figure 5B–D), similarly to the expression of AhR, a major receptor for endogenous KYN signaling. Analysis of the associations between PTH1R, AhR and the genes involved in osteogenesis revealed that both PTH1R- and AhR-dependent signaling could affect the expression of genes involved in the proliferation/early stage of differentiation of both preosteoblasts and preosteoclasts, such as ATF4, Forkhead box transcription factor 1 (FOXO1), cyclin E1 and RANK. However, it seems that OB differentiation was more prone to PTH1R-dependent signaling, whereas OC differentiation was mediated by AhR activation (Table 1). Moreover, as shown in Table 2, there were close interrelationships between the expression of the genes involved in osteoblastogenesis, as well as between these genes and RANK expression. These results indicate that the early stages of osteoblasto- and osteoclastogenesis are tightly coupled with each other, and what is important, they may be regulated by both bone KYN and serum PTH, as PTH1R and AhR are expressed in both OBs and OCs [27,34].

In subsequent analyses, we wanted to check whether the activation of the KYN pathway in bone tissue may affect osteogenesis, and whether this mechanism can impact cAMP levels and bone turnover. As presented in Table 3, activation of the KYN system in trabecular bone tissue was associated with stronger expression of the genes involved in osteoblastogenesis, particularly with ATF4 and cyclin D1. However, intensification of osteogenesis was associated with a reduction of cAMP and bone turnover (especially bone resorption) at this bone level. These data suggest that the activation of the KYN pathway in trabecular bone, as well as AhR-mediated signaling, can amplify the differentiation of early forms of OBs and OCs, resulting in the disruption of cAMP signaling and the formation of immature cells that are unable to perform their physiological functions. This is in line with our previous study [21], in which we observed that KYN may support the early stage of osteoblast differentiation, which is particularly intensified in CKD [35]. Yamamoto et al. [36] also observed that KYN signaling through AhR maintained the undifferentiated state of human embryonic stem cells. In the existing literature, there is scarce and contradictory evidence suggesting that KYN may be important for osteogenic differentiation. El Refaey et al. [37] showed that KYN in in vitro conditions significantly inhibited bone marrow mesenchymal stem cell proliferation and differentiation into osteoblasts. A recent study of Pierce et al. [38] demonstrated that KYN suppresses osteoblastic cell energetics in vitro and osteoblast number in adult mice. Opposing results were obtained by Vidal et al. [39], who demonstrated that blocking the KYN pathway through IDO—1 inhibition led to impaired osteoblastic differentiation in vitro, and that IDO-1 deficient mice were osteopenic. In relation to the role of AhR in OBs function, it is believed that AhR agonism has dose-dependent effects on OBs, in which hyperactivation inhibits bone formation [34,40], and this is in line with our results. In the case of OCs, the dose and duration of agonist exposure are important variables in AhR-mediated modulation of OC differentiation, and the different ligands bound with AhR can play diverse roles [40].

In contrast to trabecular bone, weak but inverse relations were observed between the KYN system and the expression of the genes involved in osteogenesis at the cortical bone level. Meanwhile, we observed a positive association between ATF4 expression and BTMs, particularly TRAP-5b activity (Figure 6). Even though the critical role of ATF4 in the anabolic action of PTH on OBs and bone formation is well established [41,42], this transcription factor can also play a direct role in regulating OC differentiation [43]. It has been shown that ATF4 was crucial for the induction of RANK expression on bone marrow monocyte cultures and bones of mice with ATF4 deletion; and lack of ATF4 caused a shift in OCs precursors to macrophages [43]. Since ATF4 gene expression was strongly related to both osteoblasto- and osteoclastogenesis genes in this study (Table 2), and previously we showed that the PTH-mediated PTH1R/ATF4 axis was associated with anabolic effects in the bone of these rats [16], we suppose that ATF4 may be a PTH-dependent link, coupling bone resorption with bone formation in young, uremic organisms. Moreover, the intensification of resorptive activity was accompanied by a slight increase in bone formation, reflected by ALP activity (Figure 2D). These results are in agreement with the previous observation that PTH can promote OC activation rather than OC formation [14] and that some aspects of the resorptive action of PTH are necessary for its anabolic effects in bone [10,11,12,13]. Taking all these results together, we presume that in the conditions of inhibited KYN pathway in cortical bone, PTH regains an advantage over KYN and may exert anabolic effects via the PTH1R/ATF4 axis, which is confirmed by cAMP accumulation at this bone compartment.

To date, this is the first study examining the impact of bone KYN on bone biomechanical properties in young rats with CKD. Our previous study [19] revealed detailed changes in the results of biomechanical tests during CKD development. Biomechanical properties were determined on two different bone types: the cortical bone of the femoral diaphysis using the three-point bending test and the mixed cortico-trabecular bone using the bending test of the femoral neck. Generally, an impairment in the parameters of the femoral neck bending test was observed during CKD development (Figure 7A). However, the cortical bone strength measured by the three-point bending test of femoral diaphysis was preserved in animals with CKD, and the majority of parameters even improved during the three months of CKD progression (Figure 7B). The results of the present study indicated that the activation of the KYN pathway in trabecular bone, through a reduction of bone turnover, may unfavorably affect trabecular bone biomechanics, significantly increasing stiffness and decreasing dl (Fy)—the maximal deformation under elastic conditions (Table 4). This results in the femoral neck becoming more fragile and may be more prone to fracture. This finding is in line with the study of Herlin et al. [44], who demonstrated that the exposition of adult mice to dioxin, another AhR ligand, resulted in stiffer and mechanically weaker bones.

Opposing correlations were observed between cortical bone biomechanics and KYN, whose levels were significantly reduced during the three-month period of CKD development. Interestingly, the activation of bone resorptive activity was positively related to the majority of the analyzed biomechanical parameters. Previously, we showed that serum PTH and the bone PTH1R/ATF4 axis beneficially impacted cortical bone strength in young rats with CKD [16], and the present results further support this observation by demonstrating a link between PTH-dependent ATF4 expression and TRAP—5b activity at the cortical bone level. ATF4 gene expression has been reported both in OBs and OCs [41,42,43], and the fact that herein we observed a clear relation only between ATF4 expression and osteoclastic activity (although a positive, weak association existed between ATF4 expression and ALP activity as well) may be explained by varying temporal regulation of PTH—dependent maturation of pre-osteoblasts and pre-osteoclasts, which has been previously described by Locklin et al. [45]. Taken together, the reduction of the KYN pathway in cortical bone allowed to “unblock” PTH-dependent bone turnover, which translated into better biomechanical parameters of the cortical bone.

Some limitations should be considered in the interpretation our results. Firstly, the cross—sectional design of this study does not determine whether a causal relationship exists between bone KYN levels and bone integrity, although we proved that the varying KYN concentrations (trabecular versus cortical) may interact differently with bone turnover and strength. Secondly, we cannot exclude the possibility that the observed associations could be partially attributed to other factors that may affect bone turnover or the KYN pathway that were not considered in this study. For example, teriparatide, a recombinant human PTH, has been previously reported to increase cortisol secretion from adrenals of women treated for osteoporosis, and this has been suggested to blunt the anabolic effect of teriparatide [46]. Moreover, the use of young rats may be problematic with regard to evaluation of bone turnover. To further define the mechanisms underlying the association of CKD-induced KYN pathway activation with anabolic effect of PTH, the additional intervention studies are needed in future. The histomorphometric evaluation of bone could be also helpful in future studies to support our hypothesis. A major strength of our study is the measurement of BTMs and KYN directly in the appropriate bone tissue, as the determination of these compounds in peripheral blood may not accurately represent the bone microenvironment [16]. Furthermore, the studied groups were homogeneous with regard to age, sex, diet and the absence of medication.

This is the first study to show that the activation of the paracrine KYN pathway in bone may play a vital role in antagonizing the anabolic PTH-dependent impact on bone mediated by the PTH1R/ATF4/cAMP axis. The ability of bone KYN, through AhR signaling, to affect the expression of genes involved in the proliferation/early differentiation stages of OBs and OCs promotes disturbances in bone turnover and, consequently, bone strength in young rats with CKD. Seeing that SPHT and abnormalities in TRP metabolism via the KYN pathway are commonly observed in CKD [4,8,9,19,20], the results of this study may help to better understand the role of accumulated bone KYN in skeletal health in CKD-MBD. This issue is especially important for the pediatric population, where optimum levels for PTH management in the course of CKD presents a challenge [22,47].

## 4. Materials and Methods

### 4.1. Animals

All procedures were conducted in strict adherence to Animal Research: Reporting of In Vivo Experiments (ARRIVE) guidelines, directive 2010/63/EU of the European Parliament and of the Council on the protection of animals used for scientific purposes and the national laws and were approved by the Local Ethical Committee on Animal Testing at the Medical University of Bialystok (Permit Number: 17/2012) [19]. Special care was taken to minimize the suffering and number of animals necessary to perform the procedures. Fortyfour male, 4 weeks old Wistar rats were randomly divided into two groups in which rats were subtotal nephrectomized (CKD) and sham operated (CON) as previously described [19]. Standard rodent food and tap water were available ad libitum. 

### 4.2. Sample Preparation

Blood samples and bones were collected one month (CKD-1, CON-1) and 3 months after 5/6 nephrectomy (CKD-3, CON-3) [19]. The bone tissue was taken from the distal femoral epiphysis (trabecular bone) and femoral diaphysis (cortical bone). After weighing, the appropriate bone tissue was rinsed, homogenized in a cold potassium phosphate buffer (50 mM, pH = 7.4; Polish Chemicals Reagents, Gliwice, Poland) using a high-performance homogenizer (Ultra-Turrax T25; IKA, Staufen, Germany) equipped with a stainless steel dispersing element (S25N-8G; IKA, Staufen, Germany). Next, 10% homogenates were centrifuged at 700× *g* for 10 min at 4 °C, and then the supernatant was collected and stored at −80 °C.

### 4.3. Determination of TRP and KYN in in Bone Homogenates

The homogenate was deproteinized by 20% trichloroacetic acid in a ratio of 1:4, centrifuged at 14,000× *g* for 20 min at 4 °C and then the supernatant was collected, filtered (0.45 mm Millipore filter) and stored at −80 °C. The concentrations of TRP and KYN in trabecular and cortical bone tissue homogenates were determined by the high—performance liquid chromatography (HPLC) method (Agilent Technologies, Palo Alto, CA, USA), as previously described [21].

### 4.4. Determination of BTMs Concentrations in Bone Homogenates

ALP activity was determined using colorimetric method from Biomaxima (Lublin, Poland) and TRAP-5b activities were measured by rat-specific RatTRAP™ assay from Immunodiagnostic Systems Ltd. (Frankfurt am Main, Germany). Intra- and inter-assay coefficients of variation (CV) ranged from 2%–10%, respectively. ALP and TRAP-5b activities were adjusted for protein concentration and the TRAP-5b/ALP ratio was calculated.

### 4.5. RNA Isolation and RT—qPCR Analysis

Total RNA was isolated from femoral bone using Thermo Scientific GeneJET RNA Purification Kit (Thermo Scientific, Vilnius, Lithuania). Isolated RNA samples were quantified using a spectrophotometer (Thermo Scientific, Waltham, MA, USA) to ensure A260/280 ratios of 1.8–2.0. After the reverse transcript reaction from 1 μg of mRNA, a quantitative real-time reverse transcription polymerase chain reaction (qRT-PCR) was performed using the Stratagene Mx3005P QPCR System (Agilent Technologies, Santa Clara, CA, USA) with the SG qPCR Master Mix (2×) (EURx, Gdańsk, Poland), as has been described in detail previously [24]. Primers were designed using Primer-BLAST software. The primer sequences for genes involved in osteoblastogenesis, PTH1R and TDO were accomplished elsewhere [16,21,25]. In the present study, the expression of genes involved in osteoclastogenesis and AhR were determined. In the present study, the expression of genes involved in osteoclastogenesis and AhR were determined. The primer sequences were (5′—3′ forward, reverse): ATTGGGTCAATGATGCTTGC, CTGGAACCATCTTCTCCTCC—receptor activator of nuclear factor kappaB (RANK); AATAACCAGCCCCGTCCAAG, GGTCAGAGCTGGCTCAAAGT—nuclear factor of activated T cell cytoplasmic 1 (NFATc1); CCTGGTACGTGCTGGCTGGA, CCACGGTGATGTTCGACCGC—tartrate-resistant acid phosphatase (TRAP); ACAGTTTTCCGGCTTCTTGC, GTTCGCGTCCTTCTTCATCC—aryl hydrocarbon receptor (AhR). All results were normalized to the endogenous reference glyceraldehyde 3—phosphate dehydrogenase (GADPH).

### 4.6. Bone Biomechanical Properties Determination

The biomechanical properties of femurs were determined on the cortical bone of the femoral diaphysis using three-point bending test, and on the mixed cortico—trabecular structure of the proximal femur by the bending test of the femoral neck, as has been described in detail previously [19,20]. The testing was performed with a testing machine Zwick Roell Z.2.5 (Ulm, Germany) using testXpert II software (Ulm, Germany).

### 4.7. Statistical Analysis

The Shapiro-Wilk test of normality was used for data distribution analysis. Non-Gaussian data were shown as median and a full range. Multiple group comparisons were performed using the one-way analysis of variance, and significant differences between groups were assessed by the Duncan’s post-hoc test at *p* < 0.05. The correlations between study variables were calculated by Spearman’s rank correlation analysis. For statistical analysis, we used Statistica ver. 13 computer software (StatSoft, Tulsa, OK, USA). Graphic design presentation of the results was performed using GraphPad Prism 6.0 software, San Diego, CA, USA).

## Figures and Tables

**Table 1:** The association between the expression of genes involved in osteogenesis and PTH1R and AhR mRNA levels.

	FOXO1	ATF4	Cyclin D1	Cyclin E1	RUNX2	ALP	RANK	NFATc1	TRAP
PTH1R	**R = 0.805** ***p* < 0.001**	**R = 0.826** ***p* < 0.001**	**R = 0.616** ***p* = 0.002**	**R = 0.807** ***p* < 0.001**	**R = 0.829** ***p* < 0.001**	**R = 0.823** ***p* < 0.001**	**R = 0.795** ***p* < 0.001**	R = 0.116 NS	R = 0.057 NS
AhR	**R = 0.787** ***p* < 0.001**	**R = 0.545** ***p* = 0.010**	R = 0.079 NS	**R = 0.626** ***p* = 0.002**	***R = 0.400*** ***p = 0.065***	***R = 0.407*** ***p = 0.060***	**R = 0.732** ***p* = 0.001**	**R = 0.596** ***p* = 0.007**	**R = 0.539** ***p* = 0.017**

The statistically significant changes are bolded, and tendency to correlation are marked in italics. Abbreviations: NS = non statistically significant; ATF4 = activating transcription factor 4; FOXO1 = Forkhead box transcription factor 1; RUNX2 = Runt—related transcription factor 2; ALP = alkaline phosphatase; PTH1R = parathyroid hormone 1 receptor; AhR = aryl hydrocarbon receptor; RANK = receptor activator of nuclear factor kappaB; NFATc = nuclear factor of activated T cell cytoplasmic 1; TRAP = tartrate-resistant acid phosphatase.

**Table 2:** The interrelationship between genes involved in osteoblastogenesis and RANK gene expression.

	ALP	RUNX2	Cyclin E1	Cyclin D1	ATF4	RANK	NFATc1	TRAP
FOXO1	**R = 0.728** ***p* = 0.0001**	**R = 0.719** ***p* = 0.0003**	**R = 0.866** ***p* < 0.001**	R= 0.388 *p* = 0.072	**R = 0.772** ***p* < 0.001**	**R = 0.909** ***p* < 0.001**	R = 0.340 NS	R = 0.298 NS
Cyclin D1	**R = 0.721** ***p* = 0.0001**	**R = 0.725** ***p* = 0.0001**	**R=0.521** ***p* = 0.013**		**R = 0.681** ***p* < 0.001**	**R = 0.681** ***p* = 0.0005**	R = −0.299 NS	R = −0.389 NS
Cyclin E1	**R=0.748** ***p* < 0.001**	**R = 0.808** ***p* < 0.001**		**R = 0.521** ***p* = 0.013**	**R = 0.866** ***p* < 0.001**	**R = 0.842** ***p* < 0.001**	R = 0.289 NS	R = 0.211 NS
RUNX2	**R=0.895** ***p* < 0.001**		**R = 0.808** ***p* < 0.001**	**R = 0.725** ***p* = 0.0001**	**R = 0.877** ***p* < 0.001**	**R = 0.895** ***p* < 0.001**	R = −0.085 NS	R = −0.038 NS
ALP		**R = 0.895** ***p* < 0.001**	**R = 0.748** ***p* < 0.001**	**R = 0.721** ***p* = 0.0001**	**R = 0.872** ***p* < 0.001**	**R = 0.758** ***p* < 0.001**	R = −0.185 NS	R = −0.166 NS
RANK	**R = 0.758** ***p* < 0.001**	**R = 0.706** ***p* = 0.0002**	**R = 0.842** ***p* < 0.001**	**R = 0.389** ***p* = 0.074**	**R = 0.756** ***p* < 0.001**		R = 0.302 NS	R = 0.211 NS
NFATc1	R = −0.185 NS	R = −0.085 NS	R = 0.289 NS	R = −0.299 NS	R = 0.140 NS	R = 0.302 NS		**R = 0.699** ***p* = 0.0006**

The statistically significant changes are bolded, and tendency to correlation are marked in italics. Abbreviations: NS = non statistically significant; ATF4 = activating transcription factor 4; FOXO1 = Forkhead box transcription factor 1; RUNX2 = Runt-related transcription factor 2; ALP = alkaline phosphatase; PTH1R = parathyroid hormone 1 receptor; RANK = receptor activator of nuclear factor kappaB; NFATc = nuclear factor of activated T cell cytoplasmic 1; TRAP = tartrate-resistant acid phosphatase.

**Table 3:** The associations between the expression of genes of osteoblastogenesis and KYN pathway activation, cAMP and bone turnover markers at trabecular bone level.

Genes	KYN	KYN/TRP	cAMP	ALP	TRAP−5b
ATF4	**R = 0.465** ***p* = 0.049**	**R = 0.383** ***p* = 0.086**	**R = −0.687** ***p* = 0.002**	**R = −0.461** ***p* = 0.031**	**R = −0.597** ***p* = 0.004**
FOXO1	**R = 0.165** **NS**	**R = 0.452** ***p* = 0.046**	R = −0.275 NS	R = −0.199 NS	R = −0.336 NS
Cyclin D1	**R = 0.649** ***p* = 0.002**	**R = 0.615** ***p* = 0.004**	**R = −0.584** ***p* = 0.008**	**R = −0.435** ***p* = 0.048**	***R = −0.432*** ***p = 0.057***
Cyclin E1	R = 0.206 NS	***R = 0.427*** ***p = 0.060***	***R = −0.416*** ***p = 0.076***	R = −0.119 NS	R = −0.349 NS
RUNX2	R = 0.357 NS	***R = 0.411*** ***p = 0.071***	**R = −0.540** ***p* = 0.017**	R = −0.358 NS	**R = −0.444** ***p* = 0.049**
ALP	R = 0.367 NS	**R = 0.493** ***p* = 0.027**	**R = −0.635** ***p* = 0.003**	R = −0.336 NS	**R = −0.480** ***p* = 0.032**

The statistically significant changes are bolded, and tendency to correlation are marked in italics. Abbreviations: NS = non statistically significant; ATF4 = activating transcription factor 4; FOXO1 = Forkhead box transcription factor 1; RUNX2 = Runt-related transcription factor 2; ALP = alkaline phosphatase; TRAP−5b = tartrate-resistant acid phosphatase 5b; KYN = kynurenine; cAMP = cyclic adenosine monophosphate.

**Table 4:** The association between bone biomechanics, KYN levels and bone turnover markers in the trabecular and cortical bone compartments.

	Trabecular Bone				Cortical Bone			
	KYN	ALP	TRAP−5b	TRAP−5b/ALP	KYN	ALP	TRAP−5b	TRAP−5b/ALP
stiffness	**R = 0.458** ***p* = 0.032**	**R = −0.690** ***p* = 0.0002**	**R = −0.714** ***p* = 0.0003**	**R = −0.534** ***p* = 0.013**	**R = −0.752** ***p* < 0.0001**	R = −0.005 NS	**R = 0.591** ***p* = 0.004**	**R = 0.518** ***p* = 0.014**
Fy	R = −0.155 NS	R = 0.089 NS	R = 0.192 NS	R = 0.109 NS	R = −0.755 *p* < 0.0001	R = −0.132 NS	**R = 0.525** ***p* = 0.012**	**R = 0.570** ***p* = 0.005**
dl (Fy)	**R = −0.499** ***p* = 0.018**	**R = 0.655** ***p* = 0.007**	**R = 0.694** ***p* = 0.0005**	**R = 0.499** ***p* = 0.021**	**R = −0.467** ***p* = 0.028**	R = −0.185 NS	***R = 0.418*** ***p = 0.052***	**R = 0.514** ***p* = 0.014**
Fu	**R = 0.432** ***p* = 0.044**	**R = −0.438** ***p* = 0.037**	**R = −0.475** ***p* = 0.029**	***R = −0.394*** ***p = 0.078*** ****	**R = −0.543** ***p* = 0.009**	R = −0.078 NS	**R = 0.527** ***p* = 0.012**	**R = 0.598** ***p* = 0.003**
dl (Fu)	R = −0.004 NS	R = 0.279 NS	R = 0.364 NS	R = 0.242 NS	**R = 0.616** ***p* = 0.002**	R = 0.087 NS	**R = −0.531** ***p* = 0.011**	***R = −0.404*** ***p = 0.062*** ****

The statistically significant changes are bolded, and tendency to correlation are marked in italics. Abbreviations: NS = non statistically significant; Fy = yield load; dl (Fy) = displacement at yield load; Fu = ultimate load; dl (Fu) = displacement at ultimate load; ALP = alkaline phosphatase; TRAP−5b = tartrate-resistant acid phosphatase 5b; KYN = kynurenine.

## Data Availability

The data that support the findings of this study are available on reasonable request from the corresponding author.
